# Lessons from Mouse Models of High-Fat Diet-Induced NAFLD

**DOI:** 10.3390/ijms141121240

**Published:** 2013-10-24

**Authors:** Akinobu Nakamura, Yasuo Terauchi

**Affiliations:** 1 Division of Immunology and Metabolism, Graduate School of Medicine, Hokkaido University, Sapporo 060-8638, Japan; E-Mail: akinbo@tim.hi-ho.ne.jp; 2 Department of Endocrinology and Metabolism, Graduate School of Medicine, Yokohama City University, Yokohama 236-0004, Japan

**Keywords:** hyperglycemia, insulin resistance, insulin signaling, non-alcoholic fatty liver disease, non-alcoholic steatohepatitis, liver tumorigenesis

## Abstract

Nonalcoholic fatty liver disease (NAFLD) encompasses a clinicopathologic spectrum of diseases ranging from isolated hepatic steatosis to nonalcoholic steatohepatitis (NASH), the more aggressive form of fatty liver disease that may progress to cirrhosis and cirrhosis-related complications, including hepatocellular carcinoma. The prevalence of NAFLD, including NASH, is also increasing in parallel with the growing epidemics of obesity and diabetes. However, the causal relationships between obesity and/or diabetes and NASH or liver tumorigenesis have not yet been clearly elucidated. Animal models of NAFLD/NASH provide crucial information, not only for elucidating the pathogenesis of NAFLD/NASH, but also for examining therapeutic effects of various agents. A high-fat diet is widely used to produce hepatic steatosis and NASH in experimental animals. Several studies, including our own, have shown that long-term high-fat diet loading, which can induce obesity and insulin resistance, can also induce NASH and liver tumorigenesis in C57BL/6J mice. In this article, we discuss the pathophysiology of and treatment strategies for NAFLD and subsequent NAFLD-related complications such as NASH and liver tumorigenesis, mainly based on lessons learned from mouse models of high-fat diet-induced NAFLD/NASH.

## 1. Introduction

Nonalcoholic fatty liver disease (NAFLD) is histologically characterized by more than 5% hepatic triglyceride accumulation, resulting in steatosis and hepatic inflammation [1]. NAFLD encompasses a clinicopathologic spectrum of diseases ranging from isolated hepatic steatosis to nonalcoholic steatohepatitis (NASH), the more aggressive form of fatty liver disease that may progress to cirrhosis and cirrhosis-related complications, including hepatocellular carcinoma (HCC). The prevalence of NAFLD, including NASH, is also increasing in parallel with the growing pandemic of obesity [2]. It has been estimated that as many as 30% of adults in the USA and other Western countries have NAFLD [3]; the prevalence increases to more than two-thirds in obese subjects [4]. On the other hand, NASH may be present in up to 3% of the general population [5]. In addition to hepatic complications, patients with NAFLD are at an increased risk for cardiovascular diseases [6].

The prevalence of diabetes mellitus has been rising globally over the past about 30 years [7]. Today, there are an estimated 371 million adults with diabetes, with four out of five of these patients living in low- to middle-income countries [8]. Type 2 diabetes is a multifactorial disease caused by genetic predisposition as well as environmental factors that lead to an absolute or relative deficiency of insulin actions. Insulin resistance, one of the metabolic hallmarks of type 2 diabetes, occurs in a high percentage of patients (66%–83%) with NAFLD [9]. Conversely, NAFLD is highly prevalent (up to 70%) among patients with type 2 diabetes [10], in whom increased hepatic triglyceride accumulation occurs independently of the body mass index [11]. The presence of NAFLD in individuals with type 2 diabetes appears to be a risk factor for increased mortality, with the most common causes of death being malignancy and liver disease [12]. On the other hand, the presence of type 2 diabetes is not only a risk factor for the development of NASH, but also a risk factor for the development of cirrhosis and HCC [13,14]. Thus, NAFLD and type 2 diabetes frequently coexist, sharing pathogenetic abnormalities such as obesity and insulin resistance.

In this article, we discuss the pathophysiology of and treatment strategies for NAFLD and subsequent NAFLD-related complications such as NASH and liver tumorigenesis, mainly based on lessons learned from mouse models of high-fat (HF) diet-induced NAFLD/NASH.

## 2. Mouse Models of High-Fat Diet-Induced NAFLD/NASH

Animal models of NAFLD/NASH provide crucial information, not only for elucidating the pathogenesis of NAFLD/NASH, but also for examining the therapeutic effects of various agents. These animal models need to correctly reflect both the histopathology and pathophysiology of human NAFLD/NASH. The “two-hit” hypothesis proposed by Day *et al*. [15] in relation to the pathogenesis of NAFLD/NASH is widely accepted; the first hit causes fat accumulation in the hepatocytes, and the second hit causes inflammation and fibrosis. Fat accumulation in the liver is closely associated with metabolic derangements related to obesity and insulin resistance [16]. Accordingly, the livers of animal models of NASH should show steatosis, intralobular inflammation, hepatocellular ballooning, fibrosis, and ideally, hepatic tumorigenesis. Furthermore, these animals should show metabolic abnormalities such as obesity, insulin resistance and an altered adipokine profile.

Animal models of NAFLD/NASH are classified into genetically-engineered models and nutritional models. Numerous genetic models of NAFLD/NASH have been reported to date [17] In some of them, such as sterol regulatory element binding protein (SREBP)-1c-transgenic mice [18,19] and phosphatase and tensin homologue deleted on chromosome 10 (PTEN)-null mice [20], hepatic steatosis occurs first, followed subsequently by the development of steatohepatitis. On the other hand, one of the representative examples of nutritional models of NAFLD/NASH is the model established using a methionine- and choline-deficient diet (MCD model). The MCD model, which is frequently used to study more progressive liver pathologies, shows steatosis with lobular inflammation and perisinusoidal and pericentral fibrosis. However, the metabolic profile of the MCD model is opposite to that of typical human NASH, namely, this mouse model does not show obesity or insulin resistance [21,22].

A HF diet is widely used to induce hepatic steatosis and NASH in experimental animals. However, variable results are obtained with regard to the degree of steatosis, inflammation and fibrosis, and the results depend on the rodent strain, the fat content of the diet, the composition of the dietary fat, and the duration of treatment [17]. A diet enriched in fructose and cholesterol in addition to fat induced the development of NASH in C57BL/6J mice [23]; It has been shown that long-term HF diet loading can induce obesity and insulin resistance and also NASH and liver tumorigenesis in C57BL/6J mice [24,25].

**Figure 1:**
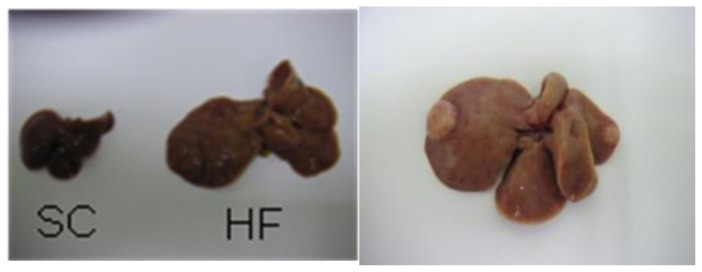
Macroscopic findings in mice fed the standard chow (SC) or high-fat (HF) diet for 60 weeks on the left, and tumors observed on the liver surface in mice fed the HF diet for 60 weeks on the right [26].

We also tested the effect of long-term HF diet loading on the development of NASH and liver tumorigenesis in C57BL/6J male mice [26]. Male littermates derived from the intercrosses were fed standard chow (SC) until 8 weeks of age, and then given free access to either SC or a HF diet. The compositions of the SC and HF diets are shown in Table 1. The fatty acid composition of the HF diet consisted of 22% saturated fatty acid (12.6% palmitic acid, 7.5% stearic acid) and 77% unsaturated fatty acid (64.3% oleic acid, 10.2% linoleic acid). The body weight, liver weight, fasting insulin level and leptin level were significantly higher, while the plasma adiponectin level was significantly lower in the mouse model fed the HF diet as compared with the findings in the mice fed SC. The glucose-lowering effect of insulin was also impaired in the HF-diet-fed mice as compared with that in the mice fed SC. Thus, long-term administration of a HF diet induced obesity and insulin resistance in the mouse model. The livers from the mice fed the HF diet for 60 weeks were enlarged as compared with those from the animals fed SC for the same duration (Figure 1). Whereas the mice fed SC showed an almost normal liver histology, those fed the HF diet exhibited the typical features of NASH in various stages of progression in the liver, including portal inflammation and blue wave-like bands of fibrotic tissue in portal lesions. The expression levels of lipogenic genes after 30 weeks and those of the genes encoding inflammatory cytokines and oxidative stress after 60 weeks were significantly increased in the HF-diet-fed mice as compared with the expression levels in the animals fed SC. Moreover, tumors of various diameters were frequently observed on the liver surface in the HF diet group (Figure 1). These nodular lesions were observed on the liver surface in 10% of the animals after 30 weeks and in 54% of the animals after 60 weeks of administration of the HF diet in the mice. In contrast, no such nodular lesions were detected in the mice that were fed SC. These results reflect the natural course of human NAFLD/NASH: in other words, steatosis developed in healthy livers, followed by an inflammatory process triggered by the release of cytokines and oxidative stress that resulted in hepatocellular degeneration, fibrosis and tumorigenesis. On the other hand, this mouse model showed some atypical features relative to cases of human NASH. Although nodular lesions were detected in these mice, fully developed cirrhosis was rarely seen. However, it has been reported that HCCs without cirrhosis are not unusual in either humans or mice, suggesting that liver cirrhosis may not be a prerequisite for the development of liver tumorigenesis, especially in the presence of NASH [24].

## 3. Role of Insulin Signaling in HF Diet-Induced NAFLD/NASH

In the liver, insulin normally suppresses hepatic glucose production by inhibiting gluconeogenesis and glycogenolysis, and promotes glycogen synthesis and lipogenesis [27]. Thus, hepatic insulin signaling is essential to the maintenance of energy homeostasis through the regulation of glucose and lipid metabolism. In the insulin-resistant state, when insulin no longer regulates carbohydrate and lipid metabolism, hyperglycemia, hepatic steatosis and dyslipidemia ensue. Hepatic insulin resistance has been classically defined as an inability of insulin to suppress the hepatic glucose output. Since insulin signaling normally induces *de novo* lipogenesis in the liver, this pathway would be expected to be impaired as well in states of insulin resistance. However, the presence of hepatic steatosis argues against this assumption, and unlike insulin signaling related to glucose homeostasis, promotion of lipogenesis by insulin is preserved, driving the synthesis and accumulation of triglyceride in the liver. One theory explaining this phenomenon has been termed “selective insulin resistance”, in which the insulin signaling pathways related to glucose metabolism are impaired, while those stimulating lipid metabolism are preserved, resulting in the co-existence of hyperglycemia and dyslipidemia in insulin-resistant states [28]. Both humans with insulin resistance caused by inherited mutations in the insulin receptor and mice with a liver-specific deletion of the insulin receptor exhibit hyperglycaemia and hyperinsulinemia, but both are protected against hepatic steatosis and hypertriglyceridemia [29,30]. This finding is consistent with the idea that not all signals are blunted in classical insulin-resistant states; rather, some signaling is preserved, particularly that related to the development of hepatic steatosis.

Insulin signaling is initiated when insulin binds to its receptor expressed on the cell membrane. The insulin receptor is a receptor tyrosine kinase, which, upon binding of insulin, is autophosphorylated and activated. Once activated, the receptor can phosphorylate tyrosine residues on the insulin receptor substrate (Irs) molecules. Irs proteins bind the phosphatidylinositol-3-kinase (PI3K) and activate it by localization to the membrane. PI3K phosphorylates the membrane lipid phosphatidylinositol 4,5-bisphosphate (PIP2) converting it into 3,4,5-trisphosphate (PIP3), an action that can be reversed by the phosphatase PTEN. PIP3 binds and localizes the 3-phosphoinositide-dependent protein kinase-1 (PDK1) to the cell membrane, along with PDK1’s targets, Akt and atypical protein kinase C (aPKC). These two target kinases are phosphorylated and activated by PDK1, eventually leading to many of the effects of insulin on glucose, lipid and protein metabolism.

Out of these molecules, Irs1 and Irs2 exhibit high structural homology, are abundantly expressed in the liver, and are thought to be responsible for transducing insulin signals from the insulin receptor to the intracellular effectors in the regulation of glucose and lipid homeostasis [31,32]. Insulin-receptor signaling can be almost exclusively mediated by Irs1 and Irs2 in the liver: Irs2 mainly functions during the fasting state and immediately after refeeding, while Irs1 functions primarily after refeeding [32]. Also, Irs1 has been observed to play a dominant role under states of nutrient excess [33].

**Figure 2:**
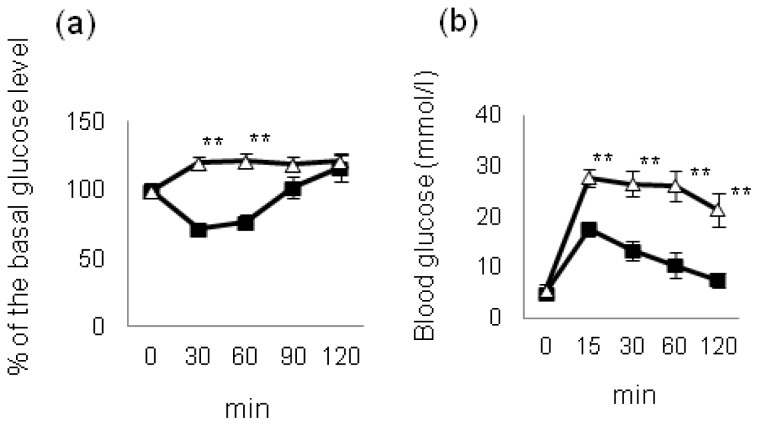
Insulin tolerance test (**a**) and oral glucose tolerance test; (**b**) in the WT and *Irs1*^−/−^ mice fed a HF diet for 30 weeks (WT: filled squares; *Irs1*^−/−^: open triangles). Values are the means ± S.E. **p < 0.01 [26].

**Figure 3:**
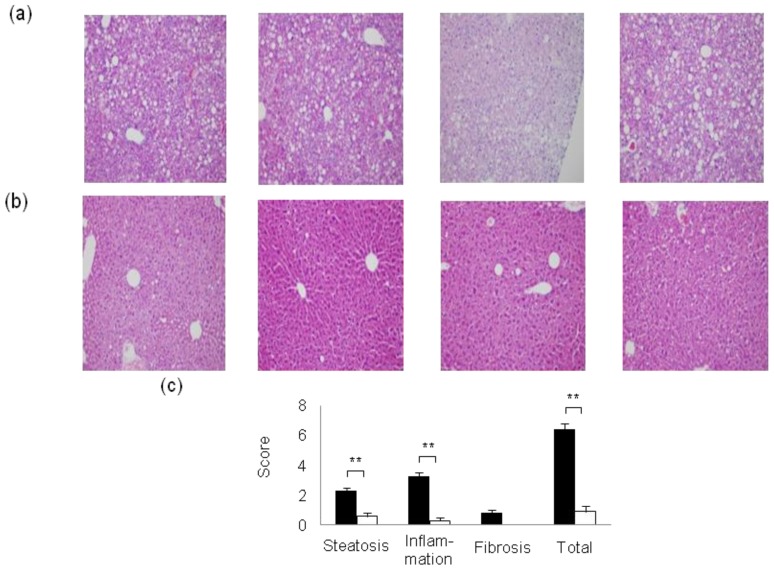
Histopathological features of livers from WT and *Irs1*^−/−^ mice fed the HF diet for 60 weeks, as assessed in H&E-stained sections ((**a**): WT; (**b**): *Irs1*^−/−^), and pathological scores for WT and *Irs1*^−/−^ mice fed the HF diet for 60 weeks; (**c**) (WT: filled bar, *Irs1*^−/−^: open bar). Values are the means ± S.E. **p < 0.01 [26].

We investigated the incidence of NASH and liver tumorigenesis in insulin receptor substrate (Irs)-1-knockout (*Irs1*^−/−^) male mice on long-term HF diet feeding [26]. *Irs1*^−/−^ mice are known to exhibit postnatal growth retardation and insulin resistance, but a normal glucose tolerance because of the compensatory beta cell hyperplasia despite resistance to the glucose-lowering effect of insulin [34–36]. Although no difference in the ratio of the visceral weight to the body weight was observed between wild-type (WT) and *Irs1*^−/−^ mice fed a HF diet, the results of the insulin tolerance test and oral glucose tolerance test revealed that the *Irs1*^−/−^ mice fed the HF diet showed severe insulin resistance and marked postprandial hyperglycemia as compared to the WT mice fed the same diet (Figure 2); the liver weight and triglyceride content of the liver were also significantly lower in the *Irs1*^−/−^ mice than in the WT mice fed the HF diet. Furthermore, although the WT mice fed the HF diet exhibited the typical features of NASH, the *Irs1*^−/−^ mice fed the HF diet showed an almost normal liver histology, with significantly lower pathological scores for NASH (Figure 3). The proportion of nodular lesions was significantly lower in the *Irs1*^−/−^ mice fed the HF diet than in the WT mice fed the same diet (9.1% in the *Irs1*^−/−^ mice *vs*. 63.6% in the WT mice). These results indicate that the disruption of Irs1 protected against HF diet-induced NASH and liver tumorigenesis despite being associated with severe hyperglycemia and insulin resistance [26].

The Irs2 levels were significantly decreased in the HF diet-fed mice as compared with those in the animals fed SC under fasting conditions, while they were similar between the two groups under refeeding conditions. Thus, insulin signaling may be decreased mainly under fasting conditions in the HF-diet group, as hyperinsulinemia associated with HF diet feeding may suppress Irs2 expression [27]. HF diet feeding might place the mice in a chronically postprandial state that preferentially inactivates Irs2, while persistent Irs1 signaling, which has been proposed as the dominant regulator of the expression of hepatic genes controlling lipogenesis, could promote lipogenesis leading to hepatic steatosis [33]. In contrast, the hepatic insulin signaling in the *Irs1*^−/−^ mice fed the HF diet was impaired, since Irs1 was absent and Irs2 signaling was suppressed by the hyperinsulinemia induced by the HF diet. Thus, the pathophysiological features in the *Irs1*^−/−^ mice fed the HF diet might be similar to those in the liver-specific Irs1/Irs2 double-knockout mice and liver-specific insulin receptor-knockout mice [30,33]. A similar situation is seen with the liver-specific loss-of-function of the p110α subunit of PI3K [37], or of Akt [38]. Since the Irs proteins lie between these steps [39], the phenotypes of the animals in these previous studies using genetically engineered insulin receptor/Irs/PI3K/Akt mouse models were probably consistent with the phenotype of the *Irs1*^−/−^ mice fed the HF diet in our study. The downstream molecular mechanisms need to be further examined in future studies.

## 4. Effect of Hyperglycemia on HF Diet-Induced NAFLD/NASH

Type 2 diabetes is a risk factor for progressive liver disease and liver disease-related death in patients with NAFLD [9]. Numerous epidemiological studies have identified associations between type 2 diabetes and several types of cancers in various populations. In particular, the strongest relationships have been demonstrated for hepatocellular carcinoma [40]. Considering the complexity of the interactions between diabetes and cancer, it is important not to overlook glucose as a potentially relevant mediator [40]. As described in the previous section, *Irs1*^−/−^ mice fed a HF diet were dramatically protected against NASH and liver tumorigenesis despite exhibiting marked postprandial hyperglycemia. These results suggest that hyperglycemia *per se* may not play a significant role in the development of NASH and liver tumorigenesis.

Glucokinase is the predominant glucose phosphorylation enzyme in the pancreatic beta cells and hepatocytes, and plays an important role as a glucose sensor in the pancreatic beta cells and as a glucose metabolism regulator in the liver [41,42]. We demonstrated that WT mice fed a HF diet maintained near-normal glucose tolerance, whereas mice with haploinsufficiency of beta cell-specific glucokinase (*Gck*+/−) developed diabetes because of insufficient beta cell hyperplasia, despite the two groups of animals showing a similar degree of insulin resistance [43–45]. These results suggest that *Gck*+/− mice fed a HF diet represent a good animal model of hyperglycemia. Thus, we investigated the effect of long-term HF diet feeding on the development of NASH and liver tumorigenesis using *Gck*+/− mice to clarify whether hyperglycemia *per se* may be involved in the pathogenesis of these conditions. The livers from the *Gck*+/− mice fed a HF diet for 60 weeks were enlarged, as compared with those from the animals fed SC for the same duration. Whereas the *Gck*+/− mice fed SC showed an almost normal liver histology, the livers of the mice fed the HF diet exhibited the typical features of NASH. The expression levels of the genes encoding inflammatory cytokines and oxidative stress were significantly elevated in the *Gck*+/− mice fed the HF diet for 60 weeks, as compared with the levels in the animals fed SC for the same duration. Moreover, nodular lesions were observed in 9.1% of the animals after 30 weeks and in 45.1% of the animals after 60 weeks of administration of a HF diet in the *Gck*+/− mice, while no such nodular lesions were detected after any length of time in the mice fed SC. Therefore, the same degrees of NASH and liver tumorigenesis were observed in the *Gck*+/− mice and WT mice fed a HF diet. These results indicate that hyperglycemia *per se* had no effect on the HF diet-induced NASH or liver tumorigenesis. Rather, hyperinsulinemia seems to play an important role in the pathogenesis of these conditions, since plasma insulin levels were almost the same between the WT mice and *Gck*+/− mice fed a HF diet. We also demonstrated previously that hyperinsulinemia, but not hyperglycemia, was associated with the severity of NASH in human subjects [46]. Moreover, data from large randomized controlled trials of aggressive glycemic control suggest that cancer risk is not reduced through improvement of glycemic control in type 2 diabetic patients [47]. Our experimental results were consistent with these epidemiological data. Recently, Johnson *et al*. advocated the hypothesis that hyperinsulinemia, but not hyperglycemia, is causally linked to an increased cancer risk [48], and our results support their hypothesis.

## 5. Impact of Current Treatments on HF Diet-Induced NAFLD/NASH

### 5.1. Lifestyle Modification

Animal models of NAFLD/NASH provide crucial information, not only for elucidating the pathogenesis of NAFLD/NASH, but also for examining therapeutic effects of various agents. Lifestyle-induced weight loss such as by caloric restriction improves the insulin sensitivity and is the ideal treatment for NAFLD/NASH. A ≥ 5% weight loss improved hepatic steatosis and a ≥ 7% weight loss also improved histological disease activity [49]. To mimic the human disease situation, we compared the C57BL/6J male mice fed the HF diet for 30 weeks followed by SC for the subsequent 30 weeks, with mice from the same genetic background that were fed the HF diet for the entire 60 weeks [26]. The mice that were switched from the HF diet to SC showed a significantly lower body weight and visceral fat weight than the animals that were fed the HF diet for the entire 60 weeks, although there were no differences in the fed-state blood glucose levels between the two groups. The diet switch improved the degree of hyperinsulinemia and insulin resistance. As compared with the findings in the mice that were continued on the HF diet for the entire 60 weeks, those that were switched from the HF diet to SC showed an almost normal liver histology. Also, the expression levels of the inflammatory cytokine- and oxidative stress-related genes were decreased significantly in the group in which the diets were switched. Moreover, the proportion of nodular lesions was also significantly decreased in the group that underwent the dietary switch (13.3% in the group in which the diets were switched *vs*. 66.7% in the group in which the HF diet was continued). These findings suggest that correction of the nutrient condition improved obesity and the related insulin resistance and protected the animals against HF diet-induced NAFLD/NASH and liver tumorigenesis. These findings were consistent with Hill-Baskin’s report [24]. Recently, it has been reported that withdrawal of the dietary overload by switching to a low-fat chow diet resulted in not only reversal of the hepatic steatosis, but also restoration of the metabolite profiles in the liver [50].

### 5.2. Insulin-Sensitizers: Thiazolidinediones

From the results observed in long-term HF diet-induced NAFLD/NASH mouse models, it was considered that the prevention of hyperinsulinemia using insulin-sensitizers such as thiazolidinediones and metformin could be a useful treatment strategy for protecting against NAFLD/NASH and liver tumorigenesis. Thiazolidinediones are peroxisome proliferator-activated receptor (PPAR) γ agonists that redistribute fat from the muscle and liver to the peripheral adipose tissue and thereby improve insulin resistance. A meta-analysis of the results of clinical trials of thiazolidinediones demonstrated an overall histological improvement of steatosis and inflammation, but not of fibrosis in patients with NASH [51]. Also, a recent nationwide case-control study revealed that the use of thiazolidinediones was associated with a decreased incidence of liver cancer in diabetic patients [52]. Although accumulating evidence suggests that thiazolidinedione treatment reduces hepatic steatosis in humans, the drug has not been demonstrated to reduce hepatic steatosis in mouse models [50,53,54]. This difference in effect may be related to the difference in the expression of PPARγ between mice and humans. While only low levels (10%–30% of adipose tissue) of PPARγ mRNA expression have been reported in the human liver and no further increased data demonstrating the increased expression of PPARγ have been reported in human hepatic steatosis, the hepatic expression of PPARγ is markedly elevated in many rodent models of diabetes and insulin resistance with hepatic steatosis [55]. However, it has been reported that pioglitazone, one of the representative thiazolidinediones, had a hepato-protective effect by attenuating the hepatic oxidative DNA damage in a HF diet mouse model [56].

### 5.3. Insulin-Sensitizers: Metformin

Metformin, a biguanide, remains the most widely used first-line drug for the treatment of type 2 diabetes [57]; the drug exerts its effect predominantly by reducing hepatic glucose production [58]. In addition to improving the glycemic control in patients with diabetes, metformin has recently drawn attention for its potential antitumor effect [59]. Epidemiological studies have shown that metformin treatment is associated with a significantly reduced risk of cancer mortality and development [60–62], and also that metformin significantly reduces the risk of development of liver cancer in humans [62–64].

We investigated the effect of metformin on HF diet-induced NASH and liver tumorigenesis using C57BL/6J male mice [65]. Eight-week-old mice were randomly divided into three groups: a control SC group fed SC, the HF group fed a HF diet, and the HF + Met group fed a HF diet and treated with metformin; in this last group, the metformin treatment was continued for 60 weeks together with the HF diet. The HF group showed significantly higher liver weights than the SC group, while the values of these parameters were significantly decreased in the HF+Met group as compared with those in the HF group. Scoring of the pathological findings showed a significant increase in the scores for liver steatosis, inflammation and fibrosis in the HF group as compared with the scores in the SC group. The scores for inflammation and fibrosis, but not for steatosis, were significantly improved in the HF + Met group as compared with those in the HF group. Nodular lesions on the liver surface were observed in approximately 70% of the mice in the HF group, but in none of the animals of the SC group. However, metformin treatment significantly decreased the incidence of the nodular lesions. These results indicate that treatment with metformin produced partial improvement of the long-term HF diet-induced NASH and prevented liver tumorigenesis in the mice. Next, an 8-week study was performed. This study showed that short-term metformin treatment improved fat accumulation in the liver, without having any effects on the expression levels of the genes encoding lipogenic and β oxidation-related enzymes; furthermore, short-term metformin treatment also suppressed adipocyte hypertrophy and improved HF diet-induced adipose tissue inflammation.

Administration of a HF diet to the mice at least initially led to a homeostatic remodeling that promoted adipose tissue expansion in response to the energy surfeit [66]. By contrast, in the later stages of chronic HF feeding, adipose tissue remodeling was observed [66]. Under the latter condition, the inability of the adipose tissue to fully meet the demand for the additional fat storage could contribute to a lipid overflow to other organs such as the liver [67–69]. A recent study showed a strong link between the inflammatory and morphological changes in the adipose tissue and the progression of steatosis to NASH [70]. In our study, metformin initially suppressed the adipocyte hypertrophy and inflammation, and improved the fat accumulation in the liver. These results suggest that metformin may suppress the overproduction in the adipose tissue of fatty acids that flow into the liver, which could contribute to the prevention of hepatic steatosis in the HF diet-fed mice. Our study indicated that metformin suppressed HF diet-induced fat accumulation in the liver after 8 weeks of treatment and improved the inflammation in the liver after 60 weeks of treatment. These findings suggest that metformin may prevent liver tumorigenesis through suppression of the natural course of the pathologic progression of NASH in mice.

To investigate whether metformin may also prevent the development of liver tumors in a mouse model of NAFLD, we compared the findings in mice fed the HF diet for 30 weeks followed by the HF diet plus metformin for the subsequent 30 weeks, with those in mice fed the HF diet for the entire 60 weeks. The results suggest that metformin treatment was insufficient to protect against HF diet-induced liver tumorigenesis in mice that had already begun to develop NAFLD; this finding may be consistent with that of a recent meta-analysis that indicated lack of any effect of metformin in improving the liver histology in patients with NAFLD/NASH [49]. Why did the mice fed the HF diet for 30 weeks followed by HF diet plus metformin for the subsequent 30 weeks fail to prevent the HF diet-induced liver tumorigenesis? One possibility is that insulin resistance, and perhaps resulting hyperinsulinemia, could play important roles in the pathogenesis of the development of NASH and liver tumorigenesis. In fact, the glucose-lowering effect of insulin was improved in the HF + Met group as compared with that in the HF group for the entire 60 weeks. By contrast, there were no differences in the glucose-lowering effect of insulin between the mice fed the HF diet for 30 weeks followed by the HF diet plus metformin for the subsequent 30 weeks and mice fed the HF diet for the entire 60 weeks. Another possibility is that since adipose tissue remodeling had already been observed in the stages of chronic HF feeding for 30 weeks, metformin is unable to suppress overproduction in the adipose tissue of fatty acids that flowed into the liver. Consequently, metformin failed to suppress the natural course of the pathologic progression of NASH in mice. Collectively, we propose that treatment with metformin may be useful as an early intervention, that is, prior to the onset of NAFLD, to prevent liver tumorigenesis in patients with diabetes. However, further research is needed to confirm this contention.

### 5.4. Lipid-Lowering Drugs

Statins (hydroxy-methyl-glutaryl coenzyme A (HMG CoA) reductase inhibitors) are the mainstay of lipid-reducing drug therapy in patients with hyperlipidemia. Since patients with NAFLD exhibit a high risk for the development of cardiovascular disease, statins are frequently prescribed to patients with NAFLD and hyperlipidemia [71]. Although the degree of biochemical and ultrasonographic regression of NAFLD was significantly greater in patients treated with statins than in those treated with fibrates [72], a randomized controlled trial revealed no effect of statins on the liver histology [73]. Ezetimibe is a sterol absorption inhibitor that blocks Niemann-Pick C1-Like 1 (NPC1L1)-mediated cholesterol absorption in the apical brush border membrane of jejunal enterocytes [74]. In humans, in addition to the effect of lowering the serum levels of low-density lipoprotein (LDL) cholesterol [75], ezetimibe has been shown to have potential effects on liver steatosis [76] and insulin resistance [77]. In an open-label, pilot study, Yoneda *et al*. reported that ezetimibe reduced the histological severity of ballooning and fibrosis [78].

We investigated the impact of ezetimibe on the insulin sensitivity in C57BL/6 mice fed a HF diet [79]. Ezetimibe had no impact on the body weight or fat mass, but significantly decreased the liver weight, hepatic triglyceride content and hepatic cholesterol content. Along with increasing the phosphorylation level of Akt, ezetimibe ameliorated hepatic insulin resistance as evaluated by a euglycemic-hyperinsulinemic clamp study and also up-regulated hepatic SHP expression. Since our results indicated that SHP silencing mainly in the liver worsened insulin resistance and that ezetimibe protected the mice against insulin resistance caused by SHP deficiency, we suggest that ezetimibe ameliorates hepatic insulin resistance via a pathway involving SHP in HF diet-fed mice.

Although our results were consistent with those of previous studies suggesting that ezetimibe improved insulin resistance and hyperinsulinemia, combined use of this drug with other agents significantly improved the histopathological findings in a mouse model of NAFLD produced by administration of a HF diet, as compared with those in animals receiving ezetimibe monotherapy [80,81]. Nozaki, *et al*. proposed a novel combination therapy for NAFLD/NASH consisting of ezetimibe and acarbose, an α-glucosidase inhibitor [80]. This report indicated that long-term combination therapy with ezetimibe and acarbose significantly reduced the severity of steatosis and inflammation/fibrosis in the liver as compared with that observed in animals administered long-term monotherapy with either drug in the mouse model of HF diet-induced NAFLD. The combined therapy decreased the serum cholesterol level more efficiently in the presence of improved insulin sensitivity by inhibiting both intestinal cholesterol and glucose absorption, thereby suppressing the inflow of lipids into the liver. Furthermore, the combination therapy promoted the release of lipids from the liver and also the β-oxidation of lipids through the activation of liver microsomal triglyceride transfer protein (MTP) and PPAR-α1, thereby reducing the hepatic triglyceride and cholesterol stores and dramatically attenuating the pathology of NAFLD/NASH. Also, the effect of combined ezetimibe plus atorvastatin therapy was investigated in obese, insulin-resistant *Alms1* mutant (*foz/foz*) mice fed a HF diet [81]. Combined ezetimibe plus atorvastatin therapy significantly lowered the hepatic free cholesterol levels in HF-fed *foz/foz* mice with NASH. Pharmacological reduction of hepatic free cholesterol was associated with normalization of JNK activation in the hepatocytes, and decrease in the severity of liver injury, hepatocyte apoptosis, NF-κB activation, adhesion molecule expression, circulating MCP-1 levels and polymorphonuclear/macrophage infiltration, with histological reversal of steatohepatitis and liver fibrosis. These findings could lead to the exploration of combined ezetimibe-based therapy in humans with NAFLD/NASH.

## 6. Conclusions

As described in this review, mouse models of HF-diet-induced NAFLD/NASH provide a wealth of information. For example, inhibition of Irs1, which is one of the key molecules in insulin signaling, might protect against HF diet-induced NASH and liver tumorigenesis. Hyperglycaemia *per se* could not affect HF diet-induced NASH and liver tumorigenesis. Lifestyle-induced weight loss and treatment with metformin could protect the animals against HF diet-induced NAFLD/NASH and liver tumorigenesis. However, the mechanisms underlying the causal relationships between obesity or diabetes and NASH or liver tumorigenesis have not yet been clearly elucidated, although the proposed enterobacteria-mediated pathogenetic mechanism has drawn attention [82,83]. With regard to the association between metabolic disorders and cancers, the incidence of not only HCC, but also of intrahepatic cholangiocarcinoma (IH-CCA) is increasing [84]. A recent meta-analysis identified many risk factors of IH-CCA that were common to HCC, such as obesity and diabetes [85]. This study supports the presence of common mechanisms involved in the pathogenesis of primary epithelial neoplasia within the liver and has prompted hypotheses to be forwarded regarding the etiology and pathogenesis of these cancers. Further research is needed to obtain further knowledge on the molecular mechanisms and to develop better therapeutic approaches.

## Figures and Tables

**Table 1:** Compositions of standard chow (SC) and the high-fat (HF) diet [26].

	SC	HF
Moisture (%)	7.9	6.2
Crude protein (%)	23.1	25.5
Crude fat (%)	5.1	32.0
Crude fiber (%)	2.8	2.9
Crude ash (%)	5.8	4.0
Nitrogen**-**free extract (%)	55.3	29.4
Total calories (kcal/100 g)	359.0	507.6

## References

[1] Tarantino G., Savastano S., Colao A. (2010). Hepatic steatosis, low-grade chronic inflammation and hormone/growth factor/adipokine imbalance. World J. Gastroenterol.

[2] Starley B.Q., Calcagno C.J., Harrison S.A. (2010). Nonalcoholic fatty liver disease and hepatocellular carcinoma: A weighty connection. Hepatology.

[3] Chalasani N., Younossi Z., Lavine J.E., Diehl A.M., Brunt E.M., Cusi K., Charlton M., Sanyal A.J. (2012). The diagnosis and management of non-alcoholic fatty liver disease: Practice guideline by the American Gastroenterological Association, American Association for the Study of Liver Diseases, and American College of Gastroenterology. Gastroenterology.

[4] Lazo M., Clark J.M. (2008). The epidemiology of nonalcoholic fatty liver disease: A global perspective. Semin. Liver Dis.

[5] Tarantino G., Finelli C. (2013). What about non-alcoholic fatty liver disease as a new criterion to define metabolic syndrome?. World J. Gastroenterol.

[6] Bhatia L.S., Curzen N.P., Calder P.C., Byrne C.D. (2012). Non-alcoholic fatty liver disease: A new and important cardiovascular risk factor?. Eur. Heart J.

[7] Danaei G., Finucane M.M., Lu Y., Singh G.M., Cowan M.J., Paciorek C.J., Lin J.K., Farzadfar F., Khang Y.H., Stevens G.A. (2011). National, regional, and global trends in fasting plasma glucose and diabetes prevalence since 1980: Systematic analysis of health examination surveys and epidemiological studies with 370 country-years and 27 million participants. Lancet.

[8] Guariguata L. (2012). By the numbers: New estimates from the IDF Diabetes Atlas Update for 2012. Diabetes Res. Clin. Pract.

[9] Smith B.W., Adams L.A. (2010). Nonalcoholic fatty liver disease and diabetes mellitus: Pathogenesis and treatment. Nat. Rev. Endocrinol.

[10] Targher G., Bertolini L., Padovani R., Poli F., Scala L., Tessari R., Zenari L., Falezza G. (2007). Prevalence of nonalcoholic fatty liver disease and its association with cardiovascular disease among type 2 diabetic patients. Diabetes Care.

[11] Gastaldelli A., Cusi K., Pettiti M., Hardies J., Miyazaki Y., Berria R., Buzzigoli E., Sironi A.M., Cersosimo E., Ferrannini E. (2007). Relationship between hepatic/visceral fat and hepatic insulin resistance in nondiabetic and type 2 diabetic subjects. Gastroenterology.

[12] Adams L.A., Harmsen S., St. Sauver J.L., Charatcharoenwitthaya P., Enders F.B., Therneau T., Angulo P. (2010). Nonalcoholic fatty liver disease increases risk of death among patients with diabetes: A community-based cohort study. Am. J. Gastroenterol.

[13] Kawamura Y., Arase Y., Ikeda K., Seko Y., Imai N., Hosaka T., Kobayashi M., Saitoh S., Sezaki H., Akuta N. (2012). Large-scale long-term follow-up study of Japanese patients with non-alcoholic Fatty liver disease for the onset of hepatocellular carcinoma. Am. J. Gastroenterol.

[14] Wong V.W., Wong G.L., Choi P.C., Chan A.W., Li M.K., Chan H.Y., Chim A.M., Yu J., Sung J.J., Chan H.L. (2010). Disease progression of non-alcoholic fatty liver disease: A prospective study with paired liver biopsies at 3 years. Gut.

[15] Day C.P., James O.F. (1998). Steatohepatitis: A tale of two “hits”?. Gastroenterology.

[16] Masuoka H.C., Chalasani N. (2013). Nonalcoholic fatty liver disease: An emerging threat to obese and diabetic individuals. Ann. N.Y. Acad. Sci.

[17] Kanuri G., Bergheim I. (2013). *In vitro* and *in vivo* models of non-alcoholic fatty liver disease (NAFLD). Int. J. Mol. Sci.

[18] Shimomura I., Hammer R.E., Richardson J.A., Ikemoto S., Bashmakov Y., Goldstein J.L., Brown M.S. (1998). Insulin resistance and diabetes mellitus in transgenic mice expressing nuclear SREBP-1c in adipose tissue: Model for congenital generalized lipodystrophy. Genes Dev.

[19] Nakayama H., Otabe S., Ueno T., Hirota N., Yuan X., Fukutani T., Hashinaga T., Wada N., Yamada K. (2007). Transgenic mice expressing nuclear sterol regulatory element-binding protein 1c in adipose tissue exhibit liver histology similar to nonalcoholic steatohepatitis. Metabolism.

[20] Horie Y., Suzuki A., Kataoka E., Sasaki T., Hamada K., Sasaki J., Mizuno K., Hasegawa G., Kishimoto H., Iizuka M. (2004). Hepatocyte-specific Pten deficiency results in steatohepatitis and hepatocellular carcinomas. J. Clin. Invest.

[21] Rinella M.E., Elias M.S., Smolak R.R., Fu T., Borensztajn J., Green R.M. (2008). Mechanisms of hepatic steatosis in mice fed a lipogenic methionine choline-deficient diet. J. Lipid Res.

[22] Rinella M.E., Green R.M. (2004). The methionine-choline deficient dietary model of steatohepatitis does not exhibit insulin resistance. J. Hepatol.

[23] Clapper J.R., Hendricks M.D., Gu G., Wittmer C., Dolman C.S., Herich J., Athanacio J., Villescaz C., Ghosh S.S., Heilig J.S. (2013). Diet-induced mouse model of fatty liver disease and non-alcoholic steatohepatitis reflecting clinical disease progression and methods of assessment. Am. J. Physiol. Gastrointest. Liver Physiol.

[24] Hill-Baskin A.E., Markiewski M.M., Buchner D.A., Wittmer C., Dolman C.S., Herich J., Athanacio J., Villescaz C., Ghosh S.S., Heilig J.S. (2009). Diet-induced hepatocellular carcinoma in genetically predisposed mice. Hum. Mol. Genet.

[25] Van Saun M.N., Lee I.K., Washington M.K., Matrisian L., Gorden D.L. (2009). High fat diet induced hepatic steatosis establishes a permissive microenvironment for colorectal metastases and promotes primary dysplasia in a murine model. Am. J. Pathol.

[26] Nakamura A., Tajima K., Zolzaya K., Sato K., Inoue R., Yoneda M., Fujita K., Nozaki Y., Kubota K.C., Haga H. (2012). Protection from nonalcoholic steatohepatitis and liver tumorigenesis in high fat-fed insulin receptor substrate-1-knockout mice despite insulin resistance. Diabetologia.

[27] Farese R.V., Zechner R., Newgard C.B., Walther T.C. (2012). The problem of establishing relationships between hepatic steatosis and hepatic insulin resistance. Cell Metab.

[28] Brown M.S., Goldstein J.L. (2008). Selective *vs.* total insulin resistance: A pathogenic paradox. Cell Metab.

[29] Semple R.K., Sleigh A., Murgatroyd P.R., Adams C.A., Bluck L., Jackson S., Vottero A., Kanabar D., Charlton-Menys V., Durrington P. (2009). Postreceptor insulin resistance contributes to human dyslipidemia and hepatic steatosis. J. Clin. Invest.

[30] Biddinger S.B., Hernandez-Ono A., Rask-Madsen C., Haas J.T., Alemán J.O., Suzuki R., Scapa E.F., Agarwal C., Carey M.C., Stephanopoulos G. (2008). Hepatic insulin resistance is sufficient to produce dyslipidemia and susceptibility to atherosclerosis. Cell Metab.

[31] Saltiel A.R., Kahn C.R. (2001). Insulin signalling and the regulation of glucose and lipid metabolism. Nature.

[32] Kubota N., Kubota T., Itoh S., Kumagai H., Kozono H., Takamoto I., Mineyama T., Ogata H., Tokuyama K., Ohsugi M. (2008). Dynamic functional relay between insulin receptor substrate 1 and 2 in hepatic insulin signaling during fasting and feeding. Cell Metab.

[33] Guo S., Copps K.D., Dong X., Park S., Cheng Z., Pocai A., Rossetti L., Sajan M., Farese R.V., White M.F. (2009). The Irs1 branch of the insulin signaling cascade plays a dominant role in hepatic nutrient homeostasis. Mol. Cell. Biol.

[34] Tamemoto H., Kadowaki T., Tobe K., Yagi T., Sakura H., Hayakawa T., Terauchi Y., Ueki K., Kaburagi Y., Satoh S. (1994). Insulin resistance and growth retardation in mice lacking insulin receptor substrate-1. Nature.

[35] Araki E., Lipes M.A., Patti M.E., Brüning J.C., Haag B., Johnson R.S., Kahn C.R. (1994). Alternative pathway of insulin signalling in mice with targeted disruption of the *IRS-1* gene. Nature.

[36] Terauchi Y., Iwamoto K., Tamemoto H., Komeda K., Ishii C., Kanazawa Y., Asanuma N., Aizawa T., Akanuma Y., Yasuda K. (1997). Development of non-insulin-dependent diabetes mellitus in the double knockout mice with disruption of insulin receptor substrate-1 and beta cell glucokinase genes. Genetic reconstitution of diabetes as a polygenic disease. J. Clin. Invest.

[37] Chattopadhyay M., Selinger E.S., Ballou L.M., Lin R.Z. (2011). Ablation of PI3K p110-α prevents high-fat diet-induced liver steatosis. Diabetes.

[38] Leavens K.F., Easton R.M., Shulman G.I., Previs S.F., Birnbaum M.J. (2009). Akt2 is required for hepatic lipid accumulation in models of insulin resistance. Cell Metab.

[39] Kadowaki T., Ueki K., Yamauchi T., Kubota N. (2012). SnapShot: Insulin signaling pathways. Cell.

[40] Johnson J.A., Carstensen B., Witte D. (2012). Diabetes and cancer (1): Evaluating the temporal relationship between type 2 diabetes and cancer incidence. Diabetologia.

[41] Matschinsky F.M., Banting Lecture (1995). A lesson in metabolic regulation inspired by the glucokinase glucose sensor paradigm. Diabetes.

[42] Matschinsky F.M. (1998). Pancreatic beta-cell glucokinase: Closing the gap between theoretical concepts and experimental realities. Diabetes.

[43] Terauchi Y., Takamoto I., Kubota N., Matsui J., Suzuki R., Komeda K., Hara A., Toyoda Y., Miwa I., Aizawa S. (2007). Glucokinase and IRS-2 are required for compensatory beta cell hyperplasia in response to high-fat diet-induced insulin resistance. J. Clin. Invest.

[44] Takamoto I., Terauchi Y., Kubota N., Ohsugi M., Ueki K., Kadowaki T. (2008). Crucial role of insulin receptor substrate-2 in compensatory beta-cell hyperplasia in response to high fat diet-induced insulin resistance. Diabetes Obes. Metab.

[45] Nakamura A., Terauchi Y., Ohyama S., Kubota J., Shimazaki H., Nambu T., Takamoto I., Kubota N., Eiki J., Yoshioka N. (2009). Impact of small-molecule glucokinase activator on glucose metabolism and beta-cell mass. Endocrinology.

[46] Nakamura A., Yoneda M., Fujita K., Tajima K., Kikuchi K., Nakajima A., Maeda S., Terauchi Y. (2011). Impact of glucose tolerance on the severity of non-alcoholic steatohepatitis. J. Diabetes Invest.

[47] Johnson J.A., Bowker S.L. (2011). Intensive glycaemic control and cancer risk in type 2 diabetes: A meta-analysis of major trials. Diabetologia.

[48] Johnson J.A., Pollak M. (2010). Insulin, glucose and the increased risk of cancer in patients with type 2 diabetes. Diabetologia.

[49] Musso G., Cassader M., Rosina F., Gambino R. (2012). Impact of current treatments on liver disease, glucose metabolism and cardiovascular risk in non-alcoholic fatty liver disease (NAFLD): A systematic review and meta-analysis of randomised trials. Diabetologia.

[50] Radonjic M., Wielinga P.Y., Wopereis S., Kelder T., Goelela V.S., Verschuren L., Toet K., van Duyvenvoorde W., van der Werff, van der Vat B., Stroeve J.H. (2013). Differential effects of drug interventions and dietary lifestyle in developing type 2 diabetes and complications: A systems biology analysis in *LDLr*^−/−^ mice. PLoS One.

[51] Musso G., Gambino R., Cassader M., Pagano G. (2010). A meta-analysis of randomized trials for the treatment of nonalcoholic fatty liver disease. Hepatology.

[52] Chang C.H., Lin J.W., Wu L.C., Lai M.S., Chuang L.M., Chan K.A. (2012). Association of thiazolidinediones with liver cancer and colorectal cancer in type 2 diabetes. Hepatology.

[53] Kus V., Flachs P., Kuda O., Bardova K., Janovska P., Svobodova M., Jilkova Z.M., Rossmeisl M., Wang-Sattler R., Yu Z. (2011). Unmasking differential effects of rosiglitazone and pioglitazone in the combination treatment with in mice fed a high-fat diet. PLoS One.

[54] Kong H., Lee S., Shin S., Kwon J., Jo T.H., Shin E., Shim K.S., Park Y.I., Lee C.K., Kim K. (2010). Down-regulation of adipogenesis and hyperglycemia in diet-induced obesity mouse model by Aloe QDM. Biomol. Ther.

[55] Semple R.K., Chatterjee V.K., O’Rahilly S. (2006). PPAR gamma and human metabolic disease. J. Clin. Invest.

[56] Hsiao P.J., Hsieh T.J., Kuo K.K., Hung W.W., Tsai K.B., Yang C.H., Yu M.L., Shin S.J. (2008). Pioglitazone retrieves hepatic antioxidant DNA repair in a mice model of high fat diet. BMC Mol. Biol.

[57] Inzucchi S.E., Bergenstal R.M., Buse J.B., Diamant M., Ferrannini E., Nauck M., Peters A.L., Tsapas A., Wender R., Matthews D.R. (2012). Management of hyperglycemia in type 2 diabetes: A patient-centered approach: Position statement of the American Diabetes Association (ADA) and the European Association for the Study of Diabetes (EASD). Diabetes Care.

[58] Bailey C.J., Turner R.C. (1996). Metformin. N. Engl. J. Med.

[59] Jalving M., Gietema J.A., Lefrandt J.D., de Jong S., Reyners A.K., Gans R.O., de Vries E.G. (2010). Metformin: Taking away the candy for cancer?. Eur. J. Cancer.

[60] Decensi A., Puntoni M., Goodwin P., Cazzaniga M., Gennari A., Bonanni B., Gandini S. (2010). Metformin and cancer risk in diabetic patients: A systematic review and meta-analysis. Cancer Prev. Res.

[61] Zhang Z.J., Zheng Z.J., Kan H., Song Y., Cui W., Zhao G., Kip K.E. (2011). Reduced risk of colorectal cancer with metformin therapy in patients with type 2 diabetes: A meta-analysis. Diabetes Care.

[62] Noto H., Goto A., Tsujimoto T., Noda M. (2012). Cancer risk in diabetic patients treated with metformin: A systematic review and meta-analysis. PLoS One.

[63] Hassan M.M., Curley S.A., Li D., Kaseb A., Davila M., Abdalla E.K., Javle M., Moghazy D.M., Lozano R.D., Abbruzzese J.L. (2010). Association of diabetes duration and diabetes treatment with the risk of hepatocellular carcinoma. Cancer.

[64] Zhang Z.J., Zheng Z.J., Shi R., Su Q., Jiang Q., Kip K.E. (2012). Metformin for liver cancer prevention in patients with type 2 diabetes: A systematic review and meta-analysis. J. Clin. Endocrinol. Metab.

[65] Tajima K., Nakamura A., Shirakawa J., Togashi Y., Orime K., Sato K., Inoue H., Kaji M., Sakamoto E., Ito Y. (2013). Metformin prevents liver tumorigenesis induced by high-fat diet in C57Bl/6 mice. Am. J. Physiol. Endocrinol. Metab.

[66] Strissel K.J., Stancheva Z., Miyoshi H., Perfield J.W., DeFuria J., Jick Z., Greenberg A.S., Obin M.S. (2007). Adipocyte death, adipose tissue remodeling, and obesity complications. Diabetes.

[67] Tan C.Y., Vidal-Puig A. (2008). Adipose tissue expandability: The metabolic problems of obesity may arise from the inability to become more obese. Biochem. Soc. Trans.

[68] Unger R.H. (2003). The physiology of cellular liporegulation. Annu. Rev. Physiol.

[69] Wang M.Y., Grayburn P., Chen S., Ravazzola M., Orci L., Unger R.H. (2008). Adipogenic capacity and the susceptibility to type 2 diabetes and metabolic syndrome. Proc. Natl. Acad. Sci. USA.

[70] Duval C., Thissen U., Keshtkar S., Accart B., Stienstra R., Boekschoten M.V., Roskams T., Kersten S., Müller M. (2010). Adipose tissue dysfunction signals progression of hepatic steatosis towards nonalcoholic steatohepatitis in C57BL/6 mice. Diabetes.

[71] Nseir W., Mahamid M. (2013). Statins in nonalcoholic fatty liver disease and steatohepatitis: Updated review. Curr. Atheroscler. Rep.

[72] Athyros V.G., Mikhaildis D.P., Didangelos T.P., Giouleme O.I., Liberopoulos E.N., Karagiannis A., Kakafika A.I., Tziomalos K., Burroughs A.K., Elisaf M.S. (2006). Effect of multifactorial treatment on non-alcoholic fatty liver disease in metabolic syndrome: A randomized study. Curr. Med. Res. Opin.

[73] Nelson A., Torres D.M., Morgan A.E. (2009). A pilot study using simvastatin in the treatment of nonalcoholic steatohepatitis: A randomized placebo-controlled trial. J. Clin. Gastroenterol.

[74] Altmann S.W., Davis H.R., Zhu L.J., Yao X., Hoos L.M., Tetzloff G., Iyer S.P., Maguire M., Golovko A., Zeng M. (2004). Niemann-Pick C1 Like 1 protein is critical for intestinal cholesterol absorption. Science.

[75] Knopp R.H., Dujovne C.A., Beaut A.L., Lipka L.J., Suresh R., Veltri E.P., Ezetimbe Study Group (2003). Evaluation of the efficacy, safety, and tolerability of ezetimibe in primary hypercholesterolaemia: A pooled analysis from two controlled phase III clinical studies. Int. J. Clin. Pract.

[76] Browning J.D., Horton J.D. (2004). Molecular mediators of hepatic steatosis and liver injury. J. Clin. Invest.

[77] González-Ortiz M., Martínez-Abundis E., Kam-Ramos A.M., Hernández-Salazar E., Ramos-Zavala M.G. (2006). Effect of ezetimibe on insulin sensitivity and lipid profile in obese and dyslipidaemic patients. Cardiovasc. Drug Ther.

[78] Yoneda M., Fujita K., Nozaki Y., Endo H., Takahashi H., Hosono K., Suzuki K., Mawatari H., Kirikoshi H., Inamori M. (2010). Efficacy of ezetimibe for the treatment of non-alcoholic steatohepatitis: An open-label, pilot study. Hepatol. Res.

[79] Muraoka T., Aoki K., Iwasaki T., Shinoda K., Nakamura A., Aburatani H., Mori S., Tokuyama K., Kubota N., Kadowaki T. (2011). Ezetimibe decreases SREBP-1c expression in liver and reverses hepatic insulin resistance in mice fed a high-fat diet. Metabolism.

[80] Nozaki Y., Fujita K., Yoneda M., Wada K., Shinohara Y., Takahashi H., Kirikoshi H., Inamori M., Kubota K., Saito S. (2009). Long-term combination therapy of ezetimibe and acarbose for non-alcoholic fatty liver disease. J. Hepatol.

[81] Van Rooyen D.M., Gan L.T., Yeh M.M., Haigh W.G., Larter C.Z., Ioannou G., Teoh N.C., Farrell G.C. (2013). Pharmacological cholesterol lowering reverses fibrotic NASH in obese, diabetic mice with metabolic syndrome. J. Hepatol.

[82] Imajo K., Fujita K., Yoneda M., Nozaki Y., Ogawa Y., Shinohara Y., Kato S., Mawatari H., Shibata W., Kitani H. (2012). Hyperresponsivity to low-dose endotoxin during progression to nonalcoholic steatohepatitis is regulated by leptin-mediated signaling. Cell Metab.

[83] Yoshimoto S., Loo T.M., Atarashi K., Kanda H., Sato S., Oyadomari S., Iwakura Y., Oshima K., Morita H., Hattori M. (2013). Obesity-induced gut microbial metabolite promotes liver cancer through senescence secretome. Nature.

[84] Chang K.Y., Chang J.Y., Yen Y. (2009). Increasing incidence of intrahepatic cholangiocarcinoma and its relationship to chronic viral hepatitis. J. Natl. Compr. Canc. Netw.

[85] Palmer W.C., Patel T. (2012). Are common factors involved in the pathogenesis of primary liver cancers? A meta-analysis of risk factors for intrahepatic cholangiocarcinoma. J. Hepatol.

